# Diagnosing ADHD in adults: Diagnostic tools and differential diagnosis

**DOI:** 10.1192/j.eurpsy.2021.226

**Published:** 2021-08-13

**Authors:** T. Gondek

**Affiliations:** Early Career Psychiatrists Committee, European Psychiatric Association, Wroclaw, Poland

## Abstract

**Abstract Body:**

Attention-Deficit/Hyperactivity Disorder (ADHD) is a common neurodevelopmental disorder, with an estimated prevalence of 2.8% in adults. It is frequently comorbid with other mental disorders and may significantly affect global functioning, leading to stigma and social discrimination. Despite its widespread occurrence in adults, many general psychiatrists do not feel well prepared to diagnose and manage this disorder. Psychiatry training curricula rarely include rotations in specialized ADHD clinics for adults or specialized courses during residency, and in many European countries such specialized clinics for adults or the most recommended medications, are not even available. It makes the recognition and treatment of ADHD often overlooked, unless it has been diagnosed in childhood. Dr. Gondek will demonstrate the diagnostic process of ADHD in adults and main directions for differential diagnosis in cases of a complex clinical picture.

**Disclosure:**

No significant relationships.

